# Supplementary material for: A Data-Mining Approach to Identify NF-kB-Responsive microRNAs in Tissues Involved in Inflammatory Processes: Potential Relevance in Age-Related Diseases
Source: Int J Mol Sci. 2023 Mar 7;24(6):5123. doi: 10.3390/ijms24065123 (PMC10049099; doi:10.3390/ijms24065123)
Supplement: Supplementary file 1 [file ijms-24-05123-s001.zip › Supplementary Figure S1.pptx]

## Slide 1
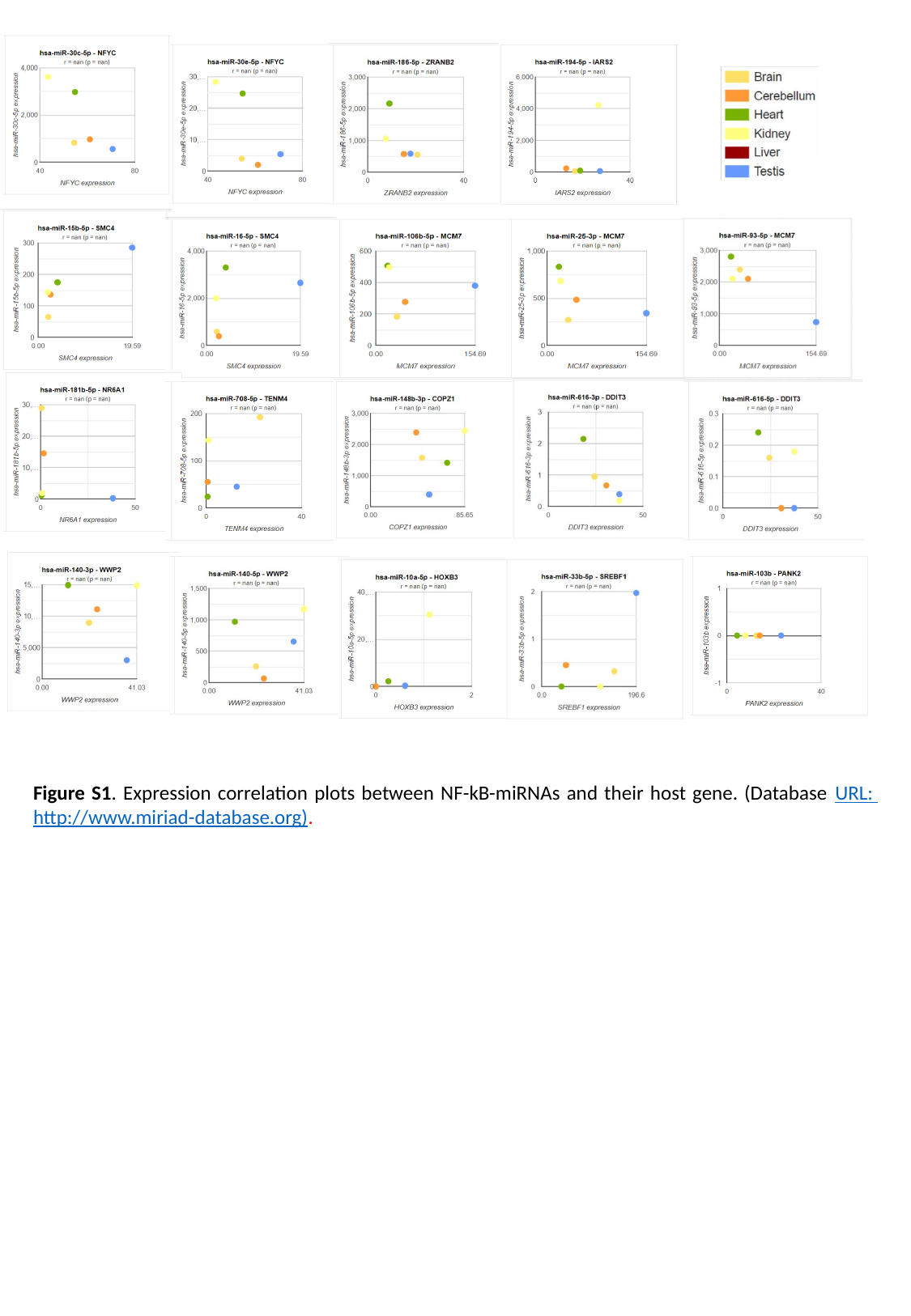

Figure S1. Expression correlation plots between NF-kB-miRNAs and their host gene. (Database URL: http://www.miriad-database.org).
